# Mycobacterial Growth Inhibition Assay (MGIA) as a Host Directed Diagnostic Tool for the Evaluation of the Immune Response in Subjects Living With Type 2 Diabetes Mellitus

**DOI:** 10.3389/fcimb.2021.640707

**Published:** 2021-05-18

**Authors:** Miriam Bobadilla-del-Valle, Francisco Leal-Vega, Pedro Torres-Gonzalez, Anabel Ordaz-Vazquez, Maria de Lourdes Garcia-Garcia, Ma. de los Angeles Tovar-Vargas, Guadalupe Delgado-Sanchez, Paola Del Carmen Guerra De Blas, Robert S. Wallis, Alfredo Ponce-De-León, José Sifuentes-Osornio

**Affiliations:** ^1^ Laboratorio de Microbiologia Clinica, Instituto Nacional de Ciencias Medicas y Nutricion Salvador Zubiran, Mexico City, Mexico; ^2^ Centro de Investigación sobre Enfermedades Infecciosas, Instituto Nacional de Salud Publica, Cuernavaca, Mexico; ^3^ LaRed- Coordinating Center, The Mexican Emerging Infectious Diseases Clinical Research Network (La Red), Mexico City, Mexico; ^4^ Aurum Institute and ACT4TB/HIV, Johannesburg, South Africa

**Keywords:** host-directed diagnostic tool, *Mycobacterium tuberculosis*, diabetes mellitus type 2 (DM2), glycemic control, mycobacterial growth inhibition assay

## Abstract

The lack of efficient and cost-effective diagnostic tools contributes to poor control of tuberculosis in endemic countries. Moreover, host biological processes influence susceptibility, and infection resolution. It is well known that comorbidities such as type 2 diabetes mellitus (DM2) affect the host immune response, making individuals more susceptible to *Mycobacterium tuberculosis* infection. Currently, there are no laboratory tools that can identify those subjects who have a higher risk of developing the disease. In this study, we used a whole blood mycobacterial growth inhibition assay to assess the immune response capacity to inhibit mycobacterial growth between healthy subjects and those living with DM2 with optimal and poor glycemic control. We also measured cytokine levels in the culture supernatant by cytokine bead arrays. We included 89 patients with DM2: 54 patients with optimal control (mean age 56.2 ± 11.75 years) and 35 patients with poor control (mean age 52.05 ± 9.94 years). We also included 44 healthy subjects as controls (mean age 42.12 ± 11.75 years). We compared the Δlog UFC (a value that represents the difference between mycobacterial growth in the control tube versus the subject’s blood) between each group. Our results demonstrate that patients with DM2 had a lower capacity to inhibit *M. tuberculosis* growth (Δlog UFC DM2 subjects 0.9581 (-0.3897 to 2.495) *vs* Δlog UFC healthy subjects 0.7190 (-0.2678 to 2.098); p=0.013). Comparing subjects living with DM2 (optimal and poor glycemic control) *vs* healthy subjects, we found only significant differences between healthy subjects and patients poorly controlled (Δlog UFC optimal control group 0.876 (-0.3897 to 2.495); Δlog UFC poor control group 1.078 (0.068 to 2.33); Δlog UFC healthy subjects 0.7190 (-0.2678 to 2.098); p= 0.022). Therefore, glycemic control assessed by glycosylated hemoglobin values influences the capacity of the host to control the infection. Our results confirm that the whole blood mycobacterial growth inhibition assay has potential utility as an *in vitro* marker of *M. tuberculosis* immunological control *in vivo* in subjects living with DM2. This assay can be used to evaluate the immune response of each individual against *M. tuberculosis*, allowing clinicians to choose a more specific host-directed therapy.

## Introduction

The lack of efficient and cost-effective diagnostic tools, therapeutics, and prevention strategies contributes to poor control of tuberculosis in endemic countries (Dye and Floyd, 2006). The World Health Organization has established that End TB targets by 2030 can only be achieved with the prevention of the progression to active tuberculosis disease among all latently infected persons (WHO, 2020).

There are three tests available for diagnosing latent TB infection: the tuberculin skin test (TST) and two types of interferon gamma release assays: QuantiFERON (Qiagen, Hilden DEU) and T-SPOT.TB (Oxford Immunotec, UK). However, none of the tests can distinguish between subjects with latent tuberculosis and active tuberculosis. Nor can those subjects with a higher risk of developing active disease be identified (Mwaba et al., 2020).

There is an online tool (TSTin3D) that estimates the lifetime risk of developing active disease based on the subject clinical profile (https://www.tstin3d.com/). However, it does not evaluate the host response of each individual against tuberculosis. It is well known that host biological processes influence susceptibility, reactivation and infection resolution (Cadena et al., 2017; Turner et al., 2017; Casadevall and Pirofski, 2018). Moreover, comorbidities such as type 2 diabetes mellitus (DM2) affect the host immune response, making individuals more susceptible to acquiring *M. tuberculosis* infection and developing disease (Restrepo and Schlesinger, 2013).

DM2 and tuberculosis are one of the most common comorbid conditions, and it has been recognized that the increase in the incidence of DM2 may contribute to tuberculosis spreading in the next years (Restrepo, 2016). Several studies have confirmed that subjects living with DM2 have a 3–6.8-fold increased risk of developing tuberculosis (Ponce-De-Leon et al., 2004; Jeon and Murray, 2008) and have more severe manifestations (Kumar et al., 2018). The host factors that influence this increased risk have been related to hyperglycemia (Mahishale et al., 2017) and altered immune response against mycobacteria, including monocytes/macrophages (Gomez et al., 2013), lymphocyte function and cytokine secretion (Vallerskog et al., 2010). Studies in mouse models infected with *M. tuberculosis* have reported an increase in the mycobacterial load and in the mortality of diabetic mice compared to controls (Martens et al., 2007). Another study evaluated the effect of glucose concentrations in U-933 monocyte-derived macrophages infected with *M. tuberculosis* and noninfected cells. This study showed that higher glucose levels impair the phagocytic capacity of these cells (Montoya et al., 2016).

Despite this evidence, there are no prospective studies that have determined the risk of developing active tuberculosis in subjects living with DM2. Moreover, in the most recent WHO consensus, treatment of latent tuberculosis was considered impractical due to the large number of individuals who would be exposed to the adverse effects of Isoniazid and the high cost of treatment for the health systems in countries with a high prevalence of both conditions (WHO, 2015).

Hence, there is an urgent need for diagnostic tools that can identify high-risk individuals and host biomarkers that can predict poor or positive outcomes after treatment. Recently, an *in vitro* test, named the whole blood mycobacterial growth inhibition assay (MGIA), has been used to evaluate the efficacy of different vaccines (Tanner et al., 2016; Tanner et al., 2020). This test evaluates the complex relationship between the host’s humoral and cellular factors, which play an important role in the immune response against mycobacterial infection.

In this study, we used MGIA to assess the immune response capacity to inhibit mycobacterial growth between healthy subjects and those living with DM2 under optimal glycemic control and poor control. We also determined cytokine levels in the culture supernatant to evaluate the immune response against *M. tuberculosis* in a more complete fashion.

## Methods

### Study Population

We recruited 89 patients older than 18 years old who met the diagnostic criteria of the American Association of Diabetes (ADA) from the Clinic of Diabetes cohort of the Instituto Nacional de Ciencias Médicas y Nutrición Salvador Zubirán, located in Mexico City. We included 54 patients (mean age 56.2 ± 11.75 years) classified as having optimal glycemic control (HbA1c ≤ 7%) and 35 patients (mean age 52.05 ± 9.94 years) classified as having poor glycemic control (HbA1c ≥ 8%) (Macisaac et al., 2009; Holmes et al., 2011; Perez et al., 2015; Hu et al., 2016). We also included 44 healthy subjects as controls (mean age 42.12 ± 11.75 years). This protocol was approved by the Institutional Ethics Committee (Ref. 952). After giving written informed consent for study participation, a questionnaire was administered for the collection of clinical characteristics. Blood samples were withdrawn for MGIA and HbA1c measurement.

### Whole Blood Mycobacterial Growth Inhibition Assay

Bacteriostatic activity was determined in whole blood according to the method previously described in 960/MGIT in the BACTEC platform (Becton Dickinson, MA, USA) (Wallis et al., 2001; Cheon et al., 2002; Janulionis et al., 2005; Wallis et al., 2010; Tanner, 2016). *M. tuberculosis* H37Rv was grown for 3 weeks on Lowenstein-Jensen medium instead of growth on liquid medium. Briefly, for each batch of reference strain stock, a standard curve was prepared that indicated the relationship between log inoculum volume and days to positivity (DTP). A titration experiment was performed in which serial 10-fold volumes of stock from 500 to 0.005 mL were inoculated into duplicate MGIT tubes to determine the general relationship between inoculum size and time-to-positivity and the specific volume predicted to be positive in 7 days. Duplicate whole-blood cultures consisted of 300 µL of heparinized blood, 300 µL of RPMI 1640 (Gibco, NY, USA) and the specified volume of mycobacterial stock for a total volume of 600 µL. RPMI was supplemented with L-glutamine and 25mM HEPES, (Corning, Manassas, VA, USA), supplemented with sodium pyruvate (Gibco™, Grand Island NY, USA), 10% fetal bovine serum (Gibco™) and essential and non-essential amino acids (SIGMA-ALDRICH, St. Louis MO, USA). Cultures were incubated at 37°C with slow constant mixing for 96 h, at which time the cells were sedimented, the liquid phase was removed, and blood cells were lysed by hypotonic lysis. Bacilli recovered after washing were inoculated into MGIT media. The log change in viability (Δlog UFC) during whole-blood culture was calculated as log(final) - log(initial), where final and initial are the volumes corresponding to the time-to-positivity values of the completed cultures and the inoculum, respectively, based on the titration curve.

### Cytokine Determination

To quantify the levels of cytokines in the culture supernatant, a BD CBA Human Th1/Th2/Th17 Cytokine Kit (BD Bioscience, San Jose, CA, USA) was used. Simultaneous detection of interleukin-2 (IL-2), interleukin-4 (IL-4), interleukin-6 (IL-6), interferon-γ (IFN-γ), tumor necrosis factor (TNF-α), interleukin-17A (IL-17A), and interleukin-10 (IL-10) was performed according to the manufacturer’s instructions. Samples were measured on a BD FACS Canto II Flow Cytometer and analyzed by FCAP ArrayTM Software (BD Bioscience).

### Statistical Analysis

#### Variable Definitions

Our dependent variable was the delta of the logarithm of CTR (Δlog UFC). Second, we considered the concentrations of the following cytokines: IL-2, IL-4, IL-6, IFN-γ, TNF-α, IL-17A and IL-10. The cytokines were measured in pg/mL.

We considered the following covariables: sex (male/female), age (years), BCG scarring (yes/no), diabetes mellitus diagnosis (yes/no), diabetes mellitus treatment, statin use (yes/no), and type of patient (healthy subjects, DM2 with optimal and poor control).

#### Analysis

The primary analysis was the estimation of changes in the log shift (Δlog UFC). If the Δlog UFC has a normal distribution, then comparison of parametric data between two groups was performed with the t-test, and for comparisons between three or more groups, ANOVA test was used.

We performed simple linear regression analyses with the independent variables. Multivariate linear regression analyses were performed to identify the factors affecting the change in Δlog UFC. We included as covariables in the adjusted model those with biological plausibility with a p-value< 0.2. In the final model, the independent variables were sex, age and type of patient.

The cytokine concentrations did not show a normal distribution; therefore, we calculated the median and interquartile range (IQR) of each cytokine by sex and type of patient. We used the Kruskal-Wallis test to evaluate the differences because it is a nonparametric test. We considered a value of p< 0.05 as statistically significant. Statistical analysis was performed using STATA^®^ 15 and GraphPad Prism v6.0 software (La Jolla, CA, United States).

### Immunophenotyping of Infected Peripheral Blood Mononuclear Cells Under Basal Conditions and With 25mM of Glucose

The PBMCs concentration was adjusted to 2x10^6^ cells/mL; 5 mL (1 x 10^7^ cells) of this suspension were seeded in supplemented RPMI medium contained in 25 mm2 cell culture flasks. RPMI was supplemented with L-glutamine and 25mM HEPES, (Corning), supplemented with sodium pyruvate (Gibco™), 10% fetal bovine serum (Gibco™) and essential and non-essential amino acids (SIGMA-ALDRICH). Cells were infected with *M. tuberculosis* H37Rv at an MOI of 0.1. Culture flasks were incubated under basal conditions and with 25mM of glucose for 48 hours. and subsequently infected

At 48 hours post-infection, the phenotypes and activation status of the helper T cells (CD4+), cytotoxic T cells (CD8+), monocytes (CD14+) were evaluated. The antibody panel was: anti-CD69-PE, anti-CD14-PE, anti-CD3-PercP, anti-CD8-PeCy7, anti-CD4-APC-Cy7, anti-CD80-APC, anti-CD86-PeCy7 (Biolegend, San Diego, CA, USA). The samples were measured using a FACSCanto II flow cytometer (BD Biosciences), and the data were analyzed using FlowJo software (Tree Star Inc., Ashland, OR, USA). The cut-off point was established using the fluorescence minus one (FMO) strategy. Single cell selection was based on cell size and granularity (FSC *vs.* SSC). First, the CD3- and CD3+ cells were selected. From the CD3+ cells, those expressing CD4 or CD8 were selected. From these the expression of the activation marker CD69+.

To identify the monocytes, cells were again selected based on size and granularity. Subsequently, the CD14+ cells were selected, and from these, the expression of co-stimulatory molecules (CD80 and C86 and HLADR) was determined.

## Results

**Figure 1:**
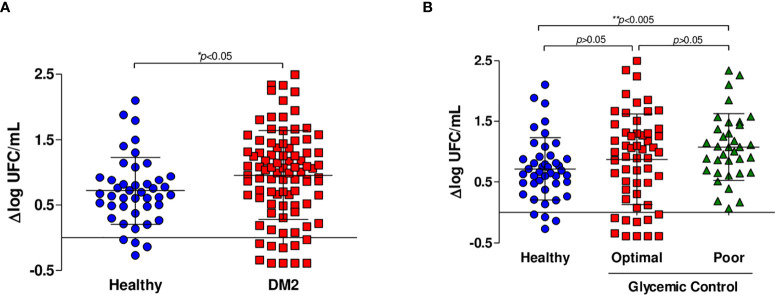
*In vitro* growth inhibition of *M. tuberculosis* H37Rv. Whole blood inhibition assays were carried out in healthy subjects (n=44), subjects with optimal glycemic control (HbA1c<7) (n=54) and subjects with poor glycemic control (Hba1c>8) (n=35). **(A)** Comparison of the capacity to inhibit mycobacterial growth of whole blood from healthy subjects (n=44) and patients living with DM2 (n=89). **(B)** Comparison of the capacity to inhibit mycobacterial growth of whole blood from healthy subjects and patients with DM2 with optimal and poor glycemic control.

To assess the immune capacity to control *M. tuberculosis* infection of subjects living with DM2 versus healthy subjects, we measured the bacteriostatic activity using MGIA as described above. In the univariate analysis, we observed that patients with DM2 had a lower capacity to inhibit *M. tuberculosis* growth (Δlog UFC DM2 subjects 0.9581 (-0.3897 to 2.495) *vs.* Δlog UFC healthy subjects 0.7190 (-0.2678 to 2.098) p=0.013) (**Figure 1A** **)**. In the simple linear regression, the association between diabetes mellitus and mycobacterial growth measured by Δlog UFC remained statistically significant (coefficient 0.239; CI 0.006-0.472, *p*=0.044) (**Table 1** **)**.

**Table 1:** Association between inhibitory capacity against mycobacterial and DM2.

Variable	n	Single linear regression				Multiple linear regression			
		Coefficient	95% Confidence interval		Valor p*	Adjusted Coefficient (n= 132)	95% Confidence interval		Valor p**
			Lower limit	Upper limit			Lower limit	Upper limit	
Male	132	-0.111	-0.337	0.116	0.336	-0.114	-0.351	0.123	0.344
Age	132	0.002	-0.007	0.012	0.631	-0.003	-0.015	0.008	0.543
BCG	122	-0.101	-3.45	0.142	0.411	-	-	-	-
Diabetes mellitus	132	0.239	0.006	0.472	**0.044**	-	-	-	-
DM treatment	132	0.204	-0.015	0.424	0.068	-	-	-	-
Statin	132	0.055	-0.177	0.288	0.637	-	-	-	-
**Type of patient**									
Healthy subject	43	Ref.				Ref.	-	-	-
DM2 with optimal control	54	0.162	-0.093	0.147	0.211	0.228	-0.799	0.536	0.145
DM2 with poor control	35	0.358	0.074	0.642	**0.014**	0.381	0.077	0.687	**0.015**

*Single linear regression; **Multiple linear regression; Bolded values represent statistically significant results.

### Glycemic Control and Control of Mycobacterial Growth

We then hypothesized that glycemic control may influence the capacity to control mycobacterial growth. When the data were analyzed comparing patients with optimal and poor control *vs.* healthy subjects, we found no significant differences between healthy subjects and patients with optimal glycemic control but did find significant differences between healthy subjects and patients with poor glycemic control (Δlog UFC optimal control group 0.876 (-0.3897 to 2.495); Δlog UFC poor control group 1.078 (0.068 to 2.33); Δlog UFC healthy subjects 0.7190 (-0.2678 to 2.098); *p*= 0.022) (**Figure 1B**). Additionally, in the multivariate linear regression analyses, poor control (those with HbA1c >8) is a factor that affected the change in Δlog UFC (coefficient 0.358; CI 0.074-0.642, *p*=0.014) (**Table 1** **)**.

### Cytokine Production

**Figure 2:**
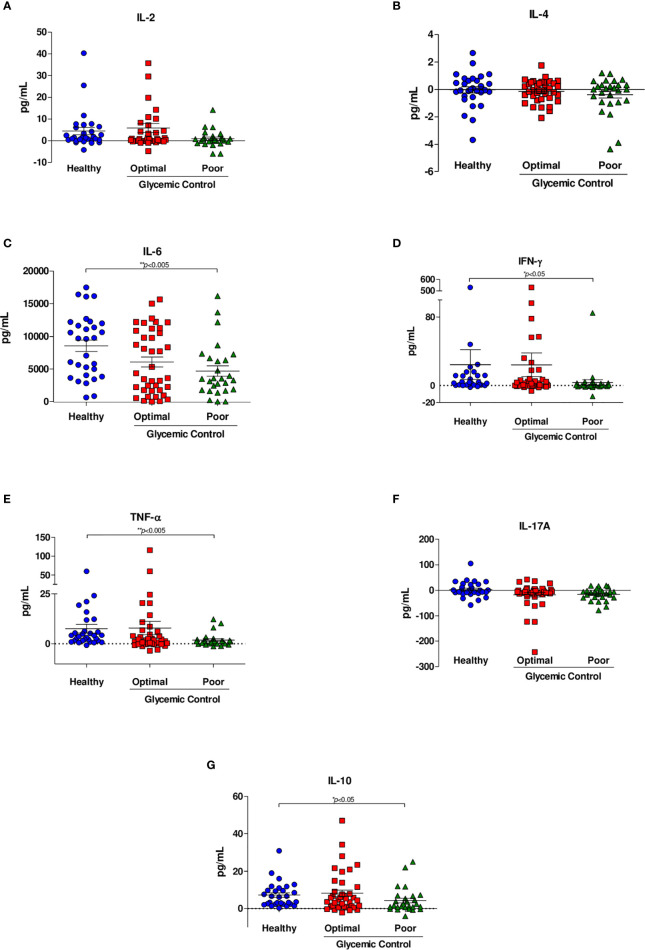
Cytokine secretion in response to *M. tuberculosis* H37Rv. Cytokine levels were assessed by CBA analyses of supernatants from whole blood inhibition assays of healthy subjects (n=44), patients with optimal glycemic control (HbA1c<7) (n=54) and patients with poor glycemic control (HbA1c>8) (n=35). **(A)** IL-2, **(B)** IL-4, **(C)** IL-6, **(D)** IFN-γ, **(E)** TNF-α, **(F)** IL-17A, **(G)** IL-10. Data are presented as scatter plots, with each symbol representing a single individual. The data were analyzed using one-way ANOVA with a *post hoc* test. *p*<0.05 was regarded as significantly different.

We measured the concentration levels of IL-2, IL-4, IL-6, IFN-γ, TNF-α, IL-17A, and IL-10 (**Table 2** **)**. No differences in the levels of the cytokines IL-2, IL-4 and IL-17A (**Figures 2A, B, F**) were observed between healthy subjects and patients with poor control. We observed a significant decrease in the level of IL-6 in patients with poor glycemic control (3789 IQR 1789-6665 *vs.* 9654 IQR 4038-12003; *p*=0.008) compared with healthy subjects (**Figure 2C** **).** The level of IFN- γ was significantly higher in healthy subjects than in patients with poor glycemic control (2.836 IQR 0.727-11.687 *vs.* 0.230 IQR -0.811-2.921; *p*=0.014) (**Figure 2D** **)**.

**Table 2:** Comparison of cytokine production (pg/mL) between studied groups.

	n	Median	IQR		*P* value
			P25	P75	
**IL-2**					
**Total**	**95**	**0.921**	**-0.112**	**4.192**	** **
Female	61	0.552	-0.120	2.396	**0.0136**
Male	34	2.047	0.112	8.301
Healthy subjects	30	1.419	0.336	6.478	0.088
DM2 with optimal control	38	0.825	-0.112	7.151
DM2 with poor control	27	0.737	-0.460	1.342
**IL-4**					
**Total**	**95**	**-0.054**	**-0.606**	**-0.504**	** **
Female	61	-0.054	-0.703	0.405	0.547
Male	34	-0.027	-0.400	0.602
Healthy subjects	30	-0.027	-0.478	0.638	0.642
DM2 with optimal control	38	-0.090	-0.606	0.449
DM2 with poor control	27	0.000	-0.903	0.316
**IL-6**					
**Total**	**95**	**5269.429**	**2432.497**	**10860.98**	
Female	61	5629.998	2476.816	10860.98	0.889
Male	34	4809.062	2432.497	10518.08
Healthy subjects	30	9654.010	4038.336	12003.25	**0.008**
DM2 with optimal control	38	3981.453	1760.912	1860.98
DM2 with poor control	27	3789.357	1789.196	6665.411
**IFN-γ**					
**Total**	**95**	**1.019**	**-0.145**	**6.496**	** **
Female	61	0.719	-0.058	4.077	0.112
Male	34	2.695	-0.145	11.687
Healthy subjects	30	2.836	0.727	11.687	**0.014**
DM2 with optimal control	38	1.099	-0.405	6.969
DM2 with poor control	27	0.230	-0.811	2.921
**TNF-α**					
**Total**	**95**	**1.675**	**0.699**	**5.056**	
Female	61	1.651	0.370	4.29	0.614
Male	34	1.736	0.299	5.98
Healthy subjects	30	3.945	1.651	6.444	**0.002**
DM2 with optimal control	38	1.544	0.087	4.799
DM2 with poor control	27	0.989	0.088	1.675
**IL-17A**					
**Total**	**95**	**-5.738**	**-15.771**	**4.419**	** **
Female	61	-5.738	-17.130	5.043	0.592

Bolded values represent statistically significant results.

Similarly, the level of TNF-α was significantly lower in patients with poor glycemic control than in healthy subjects (0.989 IQR 0.088-1.675 *vs.* 3.945 IQR 1.651-6.444; *p*=0.002) (**Figure 2E** **)**. We observed a significant decrease in the level of IL-10 in patients with poor glycemic control compared with healthy subjects (1.671 IQR 0.695-6.695 *vs* 6.345 IQR 2.438-10.995; *p*=0.047) (**Figure 2G** **)**. Finally, no significance was shown between patients with optimal glycemic control and poorly controlled or healthy and with optimal glycemic control patients in any cytokine. **Table 2** summarizes the results of each cytokine and shows that there were no differences between cytokine production and sex, with the exception of IL-2, where we observed a difference (p= 0.013).

### Effects of Glucose on the Phenotype of Infected Peripheral Blood Mononuclear Cells

Infected PBMCs that were exposed to 25 mM of glucose showed a trend of a lower percentage of T CD4+ (**Supplementary Figure 1B**), and a lower expression of the activation marker CD69+ (**Supplementary Figure 1D**). Lymphocytes T CD8+ showed no changes, however we found lower expression of the activation marker CD69 (**Supplementary Figure 1E**).

The percentage of monocytes (**Supplementary Figure 1F**) and the expression CD80 and CD86 (**Supplementary Figures 1G, H**) showed no changes. However, the expression of HLADR showed a trend to be lower in the cells cultivated under high levels of glucose (**Supplementary Figure 1I**).

## Discussion

Using MGIA, we found lower *M. tuberculosis* H37Rv growth inhibition capacity in subjects living with DM2. This failure was more remarkable in subjects living with DM2 with poor glycemic control, probably due to defective production of important cytokines, among other mechanisms involved in the control of the infection revealed in the assays that we performed. Therefore, the altered production of cytokines found in the supernatant of whole blood stimulated with *M. tuberculosis* H37Rv of patients living with DM2 supports the idea that hyperglycemia worsens the immunological response of the host. This study demonstrates for the first time that MGIA has potential utility as an *in vitro* marker of the immune response against *M. tuberculosis* infection in subjects living with DM2, defining its potential use as a host-directed diagnostic tool.

The results obtained showed that healthy subjects have a better ability to inhibit *M. tuberculosis* H37Rv than patients living with DM2. In agreement with our results, several alterations in the immune system (Kumar and Babu, 2017) and metabolic processes (Kumar et al., 2016) in these subjects living with DM2 have been described that lead to an increased risk of tuberculosis of 3–6-fold (Ponce-De-Leon et al., 2004; Jeon and Murray, 2008).

Hyperglycemia is one of the most important and obvious alterations associated with defective host responses against *M. tuberculosis*. Clinically, it has been reported that subjects living with DM2 and poor glycemic control, have a higher risk of presenting a positive sputum culture at 2–3 months, poor culture conversion and cavities (Park et al., 2012; Jiménez et al., 2013). It has been described that a hyperglycemic environment in patients living with DM2 could increase their susceptibility to pathogens due to lower interleukin production in response to infection, decreased chemotaxis and phagocytic function and leukocyte immobilization (Casqueiro et al., 2012). The MGIA confirms that patients with poor glycemic control present an alteration of the immune response that makes them less able to inhibit the growth of *M. tuberculosis* H37Rv. According to previous studies, peripheral blood monocytes from patients with DM2 had a decreased capacity to bind or ingest *M. tuberculosis* bacilli compared with patients with optimal control; these results were associated with poor glycemic control (Gomez et al., 2013). Moreover, functional changes in the macrophages of patients with DM2 have been described: reduced phagocytic capacity, decreased adhesion and chemotactic activity and reduced cytokine expression (Martinez and Kornfeld, 2014). A lower expression of costimulatory molecules has also been reported, thus affecting antigen presentation and leading to altered activation of lymphocytes, which might affect cytokine production (Lopez-Lopez et al., 2018).

This work shows that the levels of cytokines are affected in patients with DM2 and that defective production worsens in patients with poor glycemic control. In this study, we observed lower cytokine secretion of IL-6, TNF-α, IFN-γ and IL-10 in patients with poor glycemic control. In accordance with our observations, a previous study evaluated the effects of elevated glucose concentrations on the production of interleukins. PBMCs from healthy subjects exposed to high glucose levels showed suppression of the production of IL-6 and IL-10 (Reinhold et al., 1996). Kumar et al. also reported that latently infected individuals with DM2 had diminished levels of type 1 and type 17 cytokines when whole blood was stimulated with antigens of *M. tuberculosis* (Kumar et al., 2014). Therefore, this altered host response (cytokine production) could influence the susceptibility to developing tuberculosis and to having a more severe disease.

Similar to our results, in a recent study that determined the cytokine profile in patients with tuberculosis and DM, the authors observed that patients with tuberculosis and newly diagnosed DM showed low levels of proinflammatory cytokines in the plasma (Kumar et al., 2020). Cytokines are essential for the development and proliferation of the Th1 response and for the activation of cytotoxic T cells (Ayelign et al., 2019), and this defective production of these cytokines might be reflected by the marked poor response to *M. tuberculosis* infection in patients with DM2 and with poor glycemic control.

It is well known that decreased expression of IFN-γ and TNF-α increases susceptibility to tuberculosis infection (Domingo-Gonzalez et al., 2016). Additionally, the reduction in IFN-γ production impairs macrophage phagocytic activity and the antigen presentation process, thus altering intracellular bacterial persistence and providing a replication niche with clinical consequences as cavitation develops (Ayelign et al., 2019). Moreover, IL-6 is involved in the essential cellular processes of differentiation, antibody induction and effector T-cell development (Domingo-Gonzalez et al., 2016); therefore, its defective production in patients with poor glycemic control might affect the immune response against *M. tuberculosis*.

In addition, in mouse models with deficient IL-10 production, immature granulomas have been observed (Cyktor et al., 2013). Thus, considering the defect in granuloma formation as one the main containment mechanisms of transmission and severity of the disease, our data add evidence in an *ex vivo* assay about the lower production of IL-10 in patients living with DM2 and hyperglycemia.

MGIA has been used for the evaluation of the cellular and humoral immune response against *M. tuberculosis* (Tanner et al., 2016; Brennan et al., 2017; Tanner et al., 2020). Due to its technical simplicity, it is considered suitable for use in clinical studies (Brennan et al., 2017; Lee et al., 2019). For example, MGIA has been applied in clinical trials to evaluate vaccine candidates against tuberculosis. (Tanner et al., 2016; Tanner et al., 2020) demonstrated that MGIAs are useful tools in clinical studies as markers of immunity against *M. tuberculosis.*

While findings on altered cytokine production in response to *M. tuberculosis* have been consistent across studies, mainly in patients with DM2, the MGIA results of this study demonstrate that patients with DM2 and with poor glycemic control showed a lower capacity to control mycobacterial growth, presumably demonstrating that those subjects are more susceptible to developing tuberculosis; however, prospective studies are needed to validate MGIAs as a host-directed diagnostic tool. Moreover, it would be convenient to evaluate the sensitivity and specificity of this assay in a prospective cohort to confirm the potential of this screening tool to identify those subjects with increased susceptibility.

The limitation of this study is that we only evaluated cytokine production in general, and we did not evaluate other important processes, such as phagocytosis, autophagy, and apoptosis. We cannot differentiate which cytokine producer cell was affected, neither how much of the increased *M. tuberculosis* burden reported is due to intracellular replication or to extracellular multiplication. On the other hand, the strengths of this study are the model´s simplicity that permits the evaluation of a large number of subjects, and that the model is in whole blood, letting us to evaluate the global immune response capacity (including polymorphonuclear cells) to inhibit mycobacterial growth.

In order to gain insight into the effect of hyperglycemic conditions on the immune response against tuberculosis, we performed an additional experiment using PBMCS from three subjects infected *in vitro* with *M. tuberculosis*, under basal conditions, and with 25mM of glucose. We observed lower expression of the activation markers in lymphocytes and monocytes. Although these results did not show statistical significance, since it was an exploratory experiment, the reported results are consistent with previous studies (Kumar and Babu, 2017). Additional studies are needed to dissect all the immunological impaired mechanisms caused by acute and chronic hyperglycemia.

In conclusion, our results show that MGIA has potential utility as an *in vitro* marker of *M. tuberculosis* immunological control *in vivo* in patients living with DM2. This assay can be used to evaluate the immune response against *M. tuberculosis* of each host, allowing clinicians to choose a more specific directed therapy.

## Data Availability Statement

The raw data supporting the conclusions of this article will be made available by the authors, without undue reservation.

## Ethics Statement

The studies involving human participants were reviewed and approved by Comité de Ética en Investigación (Comittee on Ethics in Research) and the Comité de Investigación (Research Comittee) at the Instituto Nacional de Ciencias Médicas y Nutrición Salvador Zubirán. Written informed consent to participate in this study was provided by each patient/participant.

## Author Contributions

Study conception and design: MB-d-V, PT-G, JS-O, and APL. Laboratory work: FL-V, PT-G, and AO-V. Field data acquisition: PT-G and MT-V. Data analysis and interpretation: FL-V, PGDB, GD-S, and LG-G. Manuscript drafting: MB-d-V, FL-V, AO-V, PGDB, and GD-S. Manuscript review: RW, LG-G, JS-O, and APL. RW developed and provided the protocol for the whole blood MGIA assay. All authors contributed to the article and approved the submitted version.

## Funding

This work was supported by grant (201646) from Consejo Nacional de Ciencia y Tecnología, https://www.conacyt.gob.mx. This work was also supported by a scholarship grant (401276) from Consejo Nacional de Ciencia y Tecnología, https://www.conacyt.gob.mx/, received by FL-V, a masters student from Programa de Maestría y Doctorado en Ciencias Médicas, Odontológicas y de la Salud, Universidad Nacional Autónoma de México. The funders had no role in the study design, data collection and analysis, decision to publish, or preparation of the manuscript.

## Conflict of Interest

The authors declare that the research was conducted in the absence of any commercial or financial relationships that could be construed as a potential conflict of interest.
