# Supplementary material for: Mycobacterial Growth Inhibition Assay (MGIA) as a Host Directed Diagnostic Tool for the Evaluation of the Immune Response in Subjects Living With Type 2 Diabetes Mellitus
Source: Front Cell Infect Microbiol. 2021 May 18;11:640707. doi: 10.3389/fcimb.2021.640707 (PMC8167894; doi:10.3389/fcimb.2021.640707)
Supplement: Supplementary file 1 [file Image_1.pdf]

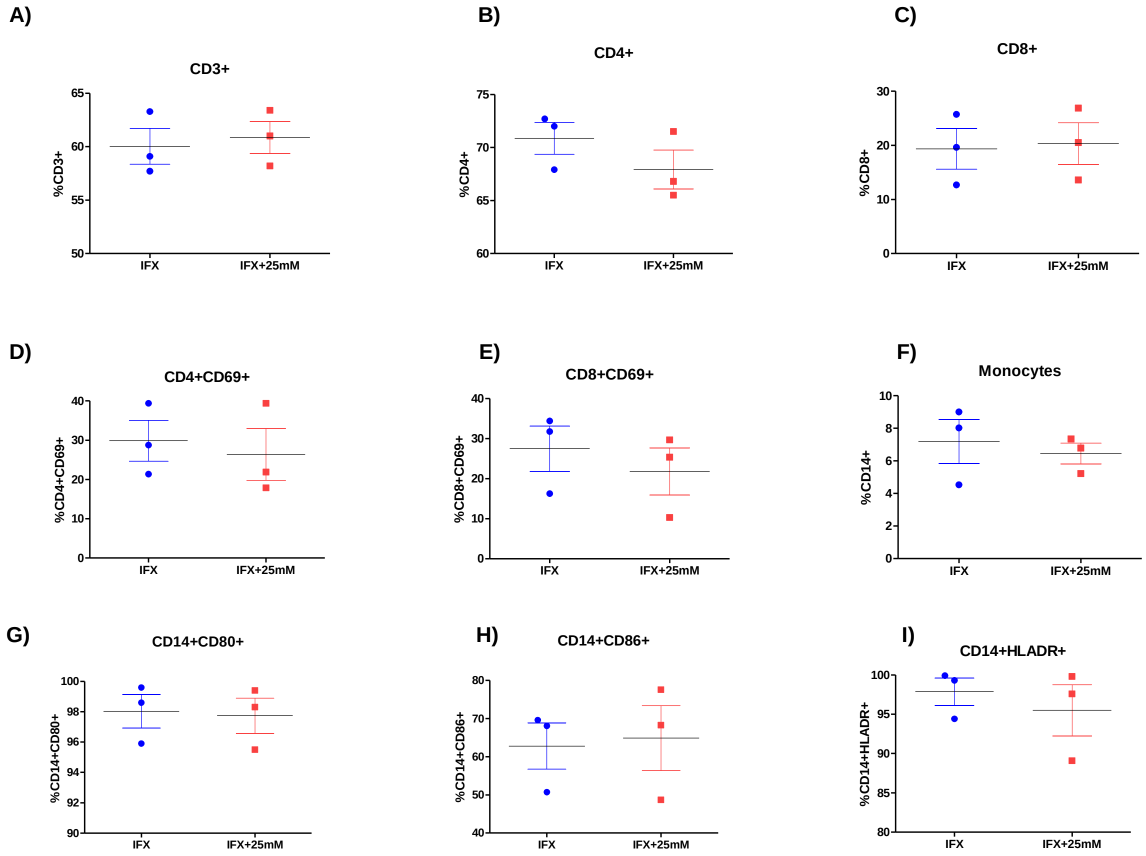

Supplementary Figure 1. Effects of glucose on the phenotype of infected peripheral blood mononuclear cells. PBMCs were infected with *M. tuberculosis* H37Rv, and incubated under basal conditions and with 25 mM of glucose. After 48 hours, the phenotypes of lymphocytes CD3+ (A), TCD4+ (B) and TCD8+ (C) lymphocytes, as well as the activation marker CD69 (D and E) were evaluated. For monocytes, we evaluated the % of CD14+ monocytes (F) and the expression of CD80 (G) and CD86 (I) is also shown, n=3. (Kruskal-Wallis)
